# An Account of a Malignant Fever, Which Appeared in the Garrison of Quebec during the Autumn of 1805, with Some Preliminary Observations on the Diseases of the Canadas

**Published:** 1806-05

**Authors:** E. Walsh


### *An Account of a malignant Fever, which appeared in the Garrison of Quebec during the Autumn of* 1805, *with some preliminary Observations on the Diseases of the Canadas.* *By* E. Walsh, M. D.

THE climate of Canada is stated, in every account of the country, as almost proverbially severe; and the extremes of heat and cold are described as meeting with scarce any intervention of moderate temperature. Such accounts are very far from being correct; yet there exists a much greater conformity of climate throughout the whole extent of the country, (now divided into the two provinces of Upper and Lower Canada) than might be supposed, in a range of 10° from north to south, and 30° from east to west.

This identity of climate may be attributed to the gradual ascent of the land towards the west, and to the great evaporation from those immense fresh water seas, which, every winter, are partly frozen, or covered with drift ice, and whose accumulated waters find their passage to the ocean, in forming the broad, deep, and rapid river, St. Laurence. They reckon, however, a month's difference of season between Quebec, in lat. 46° 47′ N. and Niagara, lat. 43° 15′ N. But this difference does not affect the general constitution of the atmosphere. The course of the winds and the seasons are equally variable in both, and the greatest summer's heat in either is about 92° of Fahrenheit; the medium 70°; whilst the mercury falls every very winter to at least 16° or 18° below zero of Farenheit at Niagara, and to 25° at Quebec. The medium heat of the year is about 55° throughout the whole extent of the Canadas.

The entire country is intersected with rivers and lakes, which have all a communication with the river St. Laurence. On the borders of these widely diffused waters the settlements are formed; the rest is primæval forest. These settlements are extremely numerous on the banks of the St. Lawrence; many of them are more than a century old.

There is no population on the face of the earth, which enjoys a greater portion of health and freedom from disease, than the inhabitants of the old settlements. Canada is indisputably the healthiest division of the Western Continent. It was the first that was colonized in North America, and the only colony of Europeans, with perhaps the exception of that recent one of New South Wales, which had not to encounter the ravages of endemic diseases.

The diseases of Canada partake of the nature of the country. They are few, but strongly marked. In Upper Canada, intermittent, remittent, and an anomalous species of the latter, called by the natives *the Lake Fever*, are prevalent. They are indeed, in most situations of the Upper Country, epidemical every autumn.

The American Physicians attempt to demonstrate, that these are gradations of one genus of fever, of which the ague and the yellow fever form the extreme links. This theory is plausible, inasmuch as the three first mentioned must be referred to one exciting cause; viz. Marsh Miasmata.

But these genera of marsh fever in the upper country are becoming, happily, every year, of less frequent occurrence; not only on account of its rapid settlement, but from a remarkable phenomenon of the lakes themselves. These vast reservoirs of fresh water are gradually but perceptibly diminishing, so that the stagnant waters of their marshy borders now find a descent, by which they are spontaneously drained into the basons of the lakes. Thus large tracks of unwholesome fens have been, and continue to be, converted, with scarce any labour, into beautiful meadows, affording luxuriant pasture to numerous herds of cattle.

It would be superfluous to describe the remittent and intermittent fevers of this country: the latter is of the tertian type, but more refractory and irregular than that usually met with in Europe; whilst the bilious remittent,

 on on the contrary, is generally milder, and yields easier to treatment than that genus of fever in tropical climates.

Some account of the Lake Fever, however, so called from being endemic only in the neighbourhood of the lakes, may not prove unnecessary. The access of this fever is not marked, like the ague and remittent fever, with strong and decisive symptoms; the diagnostics, in the beginning, are in general faint and obscure; it comes on with dull pains in the head, chills seldom increasing to rigor, lassitude, a sensation of soreness all over the body; the cuticle appears rough and dry to the feel, the tongue white but moist; the heat of the body not increased, nor does the pulse differ from that of the natural standard of health. But the most remarkable diagnostics of the lake fever are, its irregular illusive remissions, and its lengthened period of termination. By the first, the patient is induced to go about his ordinary affairs at intervals, until compelled by weakness to take to his bed again; and this occurs sometimes a few hours before he expires: otherwise, the complaint is protracted for an indefinite period, one, two, three, or four months. This fever prevails late in the autumn, and runs through half the winter; its subjects are, persons of broken constitutions, those advanced in years, and women. On the contrary, the remittent and intermittent fevers attack such as have lately arrived, with youthful and vigorous constitutions.

Marsh fevers were unusually prevalent in Upper Canada, during the summer and autumn of 1803. The grenadier company of the 49th regiment, consisting of 75 men including officers, were stationed at a block house at York. It was an advanced post, situated on the low and marshy banks of an almost stagnant river. The consequence was, that every individual of the party was attacked with fever. The post was obliged to be abandoned, nor was it until a removal to a distant part of the country that the men entirely recovered.

During the same season, numbers of people, old settlers round the bay of Quinté, one of the most fertile and populous districts in Upper Canada, died of the lake fever.

The cure of this fever is not less easy and certain at its commencement, than difficult in its advanced stages. An antimonial emetic, followed by a brisk purge, with attention to regimen for two or three days, seldom failed of curing it on the access. But if this was neglected, and the disease far advanced, such a torpor of the system was induced, as frequently rendered ineffectual the most active tive medicines. In its advanced state, after complete depletion, a remission was best obtained by sudorifics, assisted by blisters and the warm bath; after which the bark was administered in suitable forms; it generally proved inert of itself, unless assisted by aromatics and bitters. The following formula was used with much success in the hospital of Fort George.

℞. Pulv. cort. Peruviani ℥j. R. columbæ, Capsici Cayennensis sing. ʒij. Tere simul ut fiat pulvis. Drachma, ex cyatho vini lusitanic. rubri, quater indies vel sæpius sumenda.

The settlers cure marsh fevers with a fungus or agaric, which they find growing on the beech tree. It is first dried, and then rasped or grated into powder. I tried it by itself, and also joined with the bark, with decided advantage; it is an intense bitter.

From the preceding account of the lake fever, it seems to approximate to the typhus type; indeed, many practitioners of medicine settled in Canada, consider it as a typhus fever. In my opinion, however, it has a character essentially different. It is not contagious, nor did I ever meet with a case in this country of that mixed species of fever (synochus) so common in England.

In the fall of the year 1799, the Asia, a large troop ship, arrived at Quebec from the Cove of Cork, having the 41st regiment and two companies of the 6th on board. The transport was crouded, and not clean; and it was strongly suspected, that some recruits from a prison ship had introduced an infectious fever. Immediately on her arrival at Quebec, the men were transferred from the ship to batteaux in the river, the weather being raw and wet, (the last week of October); from whence they proceeded up the river to Montreal. Scarcely were they settled in quarters, when a malignant putrid fever broke out, exquisitely contagious. Numbers of the men, and several respectable inhabitants, among whom were four medical gentlemen, died of it. Several families removed from the town; and the impression it made was so forcible, that for two years afterwards the inhabitants of Montreal shunned the approach of a soldier.—This event is adduced here, as a proof how little acquainted the inhabitants of these provinces were with contagious diseases.

Quebec, indeed, being a sea port, which carries on a considerable trade with the West Indies, might doubtless be liable, as well as New York and Boston, to the consequences of imported infection. This city is built on a bold bold promontory, which projects into the river, rising 500 feet above its level. The channel, deep, clear, and rapid, sweeps round three parts of the rock, on whose shelving base the houses of the lower town are crowded, or rather huddled together. It may be easily conceived, that its streets are as filthy as possible. The upper town is more open, but not paved, which defect is partly compensated by the nature of the rock itself; it is composed of a blue schistus, (not of lime stone, as erroneously described) whose strata lie nearly perpendicular with the horizon; the consequence is, that the heaviest rains are soon absorbed.

The climate of Quebec, although in lat. 46° 47′ N. has been compared to that of St. Petersburgh in lat. 60°. but the summer season is more than a month longer. The extremes of heat and cold are, however, equally great.— The population of Quebec is computed to be between 14 and 15,000, including the garrison. Few places can boast of a greater exemption from disease; and it is remarkable, that in a climate at once so variable and severe, disorders of the chest should be of rare occurrence. Strangers, for want of precaution respecting their cloathing at the commencement of the cold season, sometimes fall victims to acute pneumonia; the natives never.

The garrison, until this autumn, enjoyed a similar degree of high health with the inhabitants. The state of the atmosphere in the summer and fall of 1805 was unusually variable; it was throughout wet, and often stormy, with the barometer remarkably low. At intervals the weather became intensely hot, the thermometer ranging during the months of July and August, and in the first week of September, between 84 and 92 deg. Early in September there was a great and sudden change, for the mercury fell to 60 deg. and about the middle of October it was as low as 40 deg. During the greater part of the two latter months, there were continued rains and storms.

During this variable season, there occurred a change of quarters of the troops in both provinces. A detachment of the 49th regiment was doing duty in Quebec; the rest of the regiment was distributed in detachments all over Upper Canada: these, therefore, had to navigate vessels and batteaux six, eight, and twelve hundred miles before they were collected at head quarters. Meanwhile, those troops already stationed at Quebec, underwent severe duty, in consequence of the increasing extent of the works, compared with the smallness of the garrison.

About the middle of August the sick list of the 49th regiment regiment, which prior to that time contained only five or six trifling cases, had increased to twenty-three, most of them fever cases; and as the detachments from the upper country arrived, it continued to increase, so that about the middle of September the number of sick in hospital amounted to sixty-five. The hospital of the 6th regiment (the greater part of which never left Quebec) was also crouded, although not in the same degree as that of the 49th.

In consequence of this unexpected increase of sick, and of some deaths, the Commandant of the forces in both Canadas ordered a medical board to be formed, to inquire into and report on the nature, cause, and present state of the fever, which then existed in the garrison.

The Board, consisting of all the medical staff officers of the garrison, and of the regimental medical officers, met accordingly; and its Report briefly stated, "That a fever, *accompanied with yellowness of the skin*, was prevalent among the troops in garrison; that this fever did not appear to have been imported, or to be infectious, but that it most probably owed its origin to a season unusually hot and moist, affecting the health of the men during a fatiguing route to Quebec, and of those also doing severe duty in garrison." The Report further stated, "That the fever was on the decline; and that as the cold weather advanced, it was expected that it would altogether disappear."

The access of this fever was sudden and violent; men were seized with it while under arms, and obliged to fall out of the ranks; chills, vertigo, and faintness were immediately succeeded by severe pains in the calves of the legs, in the loins, and in the back part of the head. The prostration of strength was so great, that the patient could not stand without support; the face was in most cases pale and contracted, although in some it was flushed, with inflamed eyes; the tongue was moist and whitish, and as the disease advanced, it was covered with a tough slough of an ash colour; in a few cases it was rough and dry, of a purple colour, with crimson edges. Although the hands felt cold to the touch, yet on pressing the pulse a burning heat might be perceived along the course of the artery; the pulse was tense and vibrated, often sunk and weak, but never quick. Towards the evening of the attack the violence of the symptoms seemed to abate, and the next morning there was an evident remission. Then, or before the second night, *the tunica conjunctiva might be observed*

*tinged*

*tinged with yellow,* which appeared to be spreading from thence to the temples and forehead. On further examination, the neck, axilla, and scrobiculus cordis betrayed also the yellow tinge. The second night proved generally restless, and on the morning of the third day there were severe exacerbations, affecting some patients with furious, others with low delirium, attended with coma. When delirium was absent, there was immense anxiety, with a continued change of posture, vomiting of every thing swallowed, and straining to vomit when there was nothing on the stomach. The matter thrown up, when it did not consist of what was taken, appeared small in quantity, frothy, and pituitous, with sometimes a greenish cast, but it was in no case dark coloured. The vomiting greatly resembled that occasioned by sea sickness. *The whole body now appeared of an intense yellow,* resembling nearest the hue of a fresh ochred wall. There was constipation of the bowels in most cases. Diarrhœa sometimes supervened, when the disease was protracted; it continued obstinate long after the fever had subsided. The urine was made in small quantities; it was of a brick colour, and clouded. In the cases which proved fatal, there was a flattering remission some hours before death; after which the pulse became intermitting; the sphincter muscles relaxed, there was involuntary discharge of the fæces and urine, and the patient calmly expired, or died comatose.

This was the course of the fever, in the greater number of cases; but in many there was considerable difference, as to the duration and succession of symptoms. In five cases there was hæmorrhage from the nose and mouth; two of the five proved fatal. In a female patient (a soldier's wife) the catamenia appeared on the access of the fever, and came on so immoderate, that means were used to restrain it. This patient died on the fourth day from the first attack. James Read, a corporal, a steady, sober man, was the first victim of this fever; towards the evening of the third day he had so complete a remission, that he talked of going to his duty the next morning; he was seized with apoplectic symptoms, however, on that morning, under which he continued in a state of insensibility, with stertorous respiration, for forty hours, and then expired. He had been bled ad ℥xxij on his admission into the hospital.

Of fifty-five cases of this fever, which occurred in the 49th regiment, six only proved fatal, and all these within the the week. No decisive crisis could be observed in those who recovered. The languor and debility continued for several weeks, and the cuticle retained its yellowness for two months. Relapses of the yellow fever are frequent in the West Indies, but no relapse took place of the fever just described. This was most probably owing to the salutary change which happened in the state of the atmosphere.

In the medical treatment of this disease, the writer of this account was not biassed by any plan of cure which he had seen adopted in the West Indies, nor by any thing he had read respecting the management of the yellow fever in the United States. He considered the fever in question as a malignant species of marsh fever, and had not that apprehension of the exhibition of antimonial emetics, in the commencement of pyrexia, which seems to have influenced, rather too much, the practice of the respectable physicians of the United States. Emetics are dreaded, it seems, as concurring with the disease in increasing the irritability of the stomach. But what remedy is so powerful in restoring the lost tone of that important organ, as the judicious employment of emetics?

In the present instance, depletion by emetics was prefered to the use of the lancet, and a full dose was found more effective than nauseating doses. Antimonii tartarazati, gr. iij. Nitrat. sodæ ℈ij. largely diluted for one dose, seldom failed to operate both ways; it always produced a moist skin and clean tongue, and in numerous instances subdued the disorder in *limine*. But when the disorder was too powerful in its access, the depletion was further pursued. Jalap and calomel in doses of twelve and five grains were administered four or five times in twenty-four hours, or oftener, till the medicine proved effective. But in the majority of cases it was found more advantageous to lose no time, but to throw in a full dose of pulv. jalapii ʒj. calomel, gr. x. ex cyatho aq. menthæ piper. fortis, which seldom failed to procure a copious alvine evacuation. Sometimes however this medicine remained inert in the stomach, in which case it was rendered active by a dose of ol. Ricini.

When the alimentary canal was effectually cleansed, a perfect remission was best obtained by sudorific medicines; and of these the aq. ammonia acetata, in which the volatile alkali was suffered just perceptibly to predominate, was proved to be, by far, the best. This medicine was administered four or five times a day, in doses suited to the state state of the patient. The diaphoresis was further assisted by the addition of fifteen or twenty drops of sp. ætheris nitrosi, and at night by the further addition of tinct. opii camphor. Bathing the lower extremities in a tepid bath was found very useful.

The most formidable symptom attendant on this fever, and the most difficult to combat, was the incessant vomiting. Great attention was required to watch a temporary cessation of this distressing symptom, and to exhibit cautiously a suitable remedy. Very small doses of a camphorated julep, with a few drops of æther; whilst some was applied externally to the pit of the stomach, frequently succeeded in soothing the perturbations of that exquisitely sensible viscus. But no bulky medicine, even an ounce of liquid; could seldom be retained. A blister applied to the epigastric region was never omitted in urgent cases, and, no doubt, proved servicable. Some of the worst cases was attended with a frightful singultus. Musk, formerly a favourite antispasmodic in fever, was thought of, but could not be procured here.

In addition to the above mode of treatment, a complete ventilation was made through the wards; the nitrous fumigation, and sprinkling the floors with vinegar, were also employed: And the affusion of cold water with vinegar, so far as it could be managed, was never neglected.

The diet, while the symptoms ran high, was very light; it consisted of tea and toast, which was prefered to every thing by the patients; sago, beef tea, &c. The drink was table beer or imperial, made palatable with essence of lemon and sugar. Sometimes cold brandy and water agreed best with the stomach.

On the first decisive remission after the fourth day, (for before then the remissions were illusory) the bark was administered; at first in decoction combined with snake-root, and as the stomach recovered its tone, in substance with an aromatic and a bitter. In the same manner wine was cautiously administered. Many of the patients took a dislike to wine; upon which, strong beer, which is brewed in this place of an excellent quality, was substituted with advantage.

Thus was this formidable and alarming fever subdued, with little comparative mortality.

In concluding this account a very few remarks may be permitted.

It is extraordinary, that the Medical Profession in the United States, and in the West Indies, should still continue, tinue, after a dozen years of enquiry and investigation, to be divided in opinion on this important question, viz. whether the yellow fever is an imported, and consequently a contagious disease? or, whether it is of spontaneous origin, and becomes endemic in certain places, from some undescribed change in the constitution of the atmosphere? However this question may be ultimately decided, there can be no doubt, that the fever above described, was neither imported nor contagious; for on the most diligent enquiry, not a single case was found to have happened among the inhabitants of the upper or lower town; and if dirt, that is, animal and vegetable substances, in various stages of putrefaction, be a prime cause of this fatal disease; then, most assuredly, no place in the world is more entitled to it than the lower town of Quebec; but, as already observed, no place of the same extent is, perhaps, so healthy.

Some formidable symptoms of the yellow fever of more southern climates, especially the black vomit, did not take place in this fever, which, on the whole, bore a milder type. It probably differs in degree only from the former, as the slow nervous fever of England differs from the putrid; and it may, therefore, not improperly, be called *typhus icteroides mitior.*

